# Adrenal crisis and mortality rate in adrenal insufficiency and congenital adrenal hyperplasia

**DOI:** 10.20945/2359-3997000000392

**Published:** 2021-07-16

**Authors:** Lia Mesquita Lousada, Berenice B. Mendonca, Tania A. S. S. Bachega

**Affiliations:** 1 Universidade de São Paulo Faculdade de Medicina Hospital das Clínicas São Paulo SP Brasil Unidade de Endocrinologia do Desenvolvimento, Laboratório de Hormônios e Genética Molecular (LIM42), Hospital das Clínicas, Faculdade de Medicina da Universidade de São Paulo, São Paulo, SP, Brasil

**Keywords:** Primary adrenal insufficiency, congenital adrenal hyperplasia, mortality, adrenal crisis, emergency care

## Abstract

Primary adrenal insufficiency (PAI) is characterized by the inability of the adrenal cortex to produce sufficient amounts of glucocorticoids and/or mineralocorticoids. Addison's disease (AD) and congenital adrenal hyperplasia (CAH) are the most frequent disorders in adults and children, respectively. Despite the diagnostic advances and the availability of glucocorticoid and mineralocorticoid replacements, adrenal crisis (AC) is still a potentially lethal condition contributing to the increased mortality, not only during the first year of life, but also throughout life. Failure in increasing glucocorticoid doses during acute stress, when greater amounts of glucocorticoids are required, can lead to AC and an increase morbimortality rate of PAI. Considering a mortality rate of 0.5 per 100 patient years, up to 1,500 deaths from AC are expected in Brazil in the coming decade, which represents an alarming situation. The major clinical features are hypotension and volume depletion. Nonspecific symptoms such as fatigue, lack of energy, anorexia, nausea, vomiting, and abdominal pain are common. The main precipitating factors are gastrointestinal diseases, other infectious disease, stressful events (e.g., major pain, surgery, strenuous physical activity, heat, and pregnancy), and withdrawal of glucocorticoid therapy. Suspected AC requires immediate therapeutic action with intravenous (iv) hydrocortisone, fluid infusion, monitoring support, and antibiotics if necessary. AC is best prevented through patient education, precocious identification and by adjusting the glucocorticoid dosage in stressor situations. The emergency card, warning about acute glucocorticoid replacement, has high value in reducing the morbidity and mortality of AC.

## INTRODUCTION

Primary adrenal insufficiency (PAI), first described by Thomas Addison in 1855, is characterized by the inability of the adrenal cortex to produce enough glucocorticoids and/or mineralocorticoids. The prevalence of PAI is around 82-144/million and the most common causes are autoimmunity (Addison's disease [AD]) in adults and genetic causes in children, especially enzymatic defects (congenital adrenal hyperplasia [CAH]) ( 1 ). These disorders are potentially life-threatening conditions due to the central role of glucocorticoids and/or mineralocorticoids in energy, salt, and fluid homeostasis ( 2 ).

Prior to Addison's time, adrenal insufficiency was an invariably fatal condition due to absence of steroid replacement therapy, with a 1-year survival rate of about 20% or less. Most patients died within the first 5 years after diagnosis. The discovery of cortisone by Hench, Kendall, and Reichstein in the late 1940s improved the survival rate dramatically. Initial data demonstrated that the life expectancy of adrenal insufficiency patients was similar to that of the general population, except when the disease was undiagnosed and patients were under poor social conditions ( 3 , 4 ).

Nevertheless, further studies among hypopituitarism patients demonstrated excessively high mortality, possibly due to inadequate glucocorticoid replacement therapy. These data stimulated additional studies about mortality rate due to adrenal insufficiency ( 5 ). Most available data came from retrospective studies. A Swedish study (n = 1,675 patients) demonstrated increased mortality in AD patients: a mortality rate 2-fold higher than that of the reference population (RR: 2.19 in males and 2.86 in females) ( 6 ). Although a Norwegian series (n = 811 patients) suggested no significant difference in mortality rate for the whole group, those patients diagnosed before 40 years old presented an increased mortality rate at 1.50 (95% CI 1.09-2.01), especially among males (2.03 [1.19-2.86]). Adrenal insufficiency was the major cause of death (15% of 130 deceased patients), most likely during adrenal crisis (AC). The mean age of death was 75.7 years for females and 64.8 years for males, representing, respectively, 3.2 and 11.2 years less than the estimated life expectancy ( 7 ).

Among children, the salt-wasting (SW) form of CAH due to 21-hydroxylase deficiency commonly presents as a neonatal SW crisis characterized by hyponatremic dehydration and, if untreated, death. Although the simple virilizing (SV) form of CAH does not usually present as a spontaneous SW crisis, special attention should be given during stressful events ( 8 ).

Prior to the introduction of newborn screening (NBS) programs for CAH, the neonatal mortality was higher, especially among SW males. This increased mortality was suggested by both the low proportion of the SW form in relation to the SV form and the low male-to-female ratio in the Hospital das Clínicas cohort, as well as in other unscreened populations ( 9 – 11 ). The CAH NBS program has been applied in Sao Paulo/Brazil since 2013. The effectiveness of this public program was demonstrated by the increasing number of male patients, reaching a similar proportion of affected males and females, as expected for an autosomal recessive disorder, and by the increasing frequency of SW in relation to SV patients (70% to 90%) ( 12 – 14 ).

Despite the earlier CAH diagnosis, through clinical means or NBS, the mortality rate remained high. Three retrospective studies comprising 1,191 English and Swedish CAH patients evidenced increased mortality in all age groups, varying from 2- to 5-fold in comparison with the general population ( 15 – 17 ).

In all these studies on mortality in adrenal insufficiency, the main causes of death were cardiovascular disease, AC, infections, and cancer. Inappropriate glucocorticoid replacement therapy, whether over- or underdosing treatment, corresponds with increased mortality in adrenal insufficiency patients. Lifelong overdosing glucocorticoid replacement therapy could be related to adverse effects such as increased frequency of cardiovascular disease, metabolic syndrome, infections, or cancer. However, insufficient glucocorticoid replacement during stress events and simultaneous illnesses can induce AC ( 6 , 7 , 18 ).

Notwithstanding the adrenal insufficiency diagnostic advances and the availability of glucocorticoid and mineralocorticoid replacements, these studies illustrate that AC is still a potentially lethal condition that contributes to the high mortality rate in adrenal insufficiency, not only during the first year of life, but also throughout life ( 15 ). In the Hospital das Clínicas cohort, 4 out of 250 classical CAH patients and 2 adult patients with bilateral adrenalectomy died due to inadequate glucocorticoid replacement during illness (unpublished data). Although there are few case reports of death due to undiagnosed adrenal insufficiency and AC in the literature ( 19 – 21 ), we hypothesize that the number of deaths from undiagnosed adrenal insufficiency and AC is higher than previously noticed due to difficulty in collecting real-world data ( 22 ). Since adrenal insufficiency and AC aren't so frequent, health professionals may not be familiar with the precocious diagnoses and treatment. These data highlight the necessity of continuous education of patients, relatives, caregivers, and physicians, emphasizing the importance of stress hydrocortisone doses during adverse events ( 23 ).

## ADRENAL CRISIS (AC)

### Pathophysiology of AC

It is well known that during stressful events, such as fever and infection, endogenous cortisol levels increase substantially in subjects with preserved adrenal function. Infection triggers the release of cytokines such as interleukin 1 (IL-1), tumor necrosis factor α (TNFα), and interleukin 6 (IL-6), which stimulate the hypothalamus-pituitary-adrenal (HPA) axis to increase the cortisol levels. As a feedback mechanism, the high glucocorticoid levels decrease the production of inflammatory cytokines to avoid exacerbated deleterious effects ( 24 ).

Patients with PAI and CAH are at risk of life-threatening AC due to their inability to intensify glucocorticoid production during stress ( 2 , 25 , 26 ). AC is more frequent in patients with PAI than in those with secondary adrenal insufficiency, possibly due to partial preservation of cortisol secretion in some patients with secondary adrenal insufficiency, as well as mineralocorticoid deficiency in those with PAI ( 26 ).

### Clinical manifestations and diagnosis of AC

Allolio and cols. defined AC, also called acute adrenal insufficiency or Addisonian crisis, as a profound impairment of general health and at least two of the following conditions: hypotension (systolic blood pressure < 100 mmHg), nausea or vomiting, severe fatigue, hyponatremia, hypoglycemia, hyperkalemia, and reversibility after administration of parenteral glucocorticoids ( 27 ).

The major clinical features of AC are volume depletion and hypotension. Volume depletion is caused by a failure to retain fluid and sodium due to the deficiency of mineralocorticoid action, which is prominent in PAI but not in secondary adrenal insufficiency. The volume depletion can be intensified by vomiting and diarrhea ( 26 , 28 ). Glucocorticoids exert a permissive effect for catecholamine action in the endothelium vessels and cardiac tissue during stress activation of the cardiovascular system. Therefore hypotension occurs secondarily to both hypovolemia and hypocortisolism ( 29 ). If hypotension first attributed to AC does not reverse after glucocorticoid parenteral infusion, the coexistence of other conditions associated with hypotension, such as sepsis, must be considered ( 26 ).

Nonspecific symptoms such as fatigue and lack of energy are common. Anorexia, nausea, vomiting, and abdominal pain are also observed in AC, being frequently misdiagnosed as gastrointestinal disease ( 25 , 27 ). Fever is frequently current, as many AC episodes are triggered by infection and due to increased inflammatory cytokines ( 30 ). At a later stage, impaired cognition and somnolence may occur ( 28 ). Hypoglycemia, more frequent in children and rare in adults, may occur due to reduced gluconeogenesis related to hypocortisolism. Cortisol deficiency may also lead to neutropenia, eosinophilia, and lymphocytosis. Combined glucocorticoid and mineralocorticoid deficiencies can result in urinary sodium loss, hyponatremia, hyperkalemia, and increased serum urea ( 2 , 26 , 31 ). Other long-term manifestations include hyperpigmentation related to hypersecretion of proopiomelanocortin-derived peptides (only in PAI), orthostatic hypotension, and, in children, failure to thrive ( 2 ).

The diagnosis of adrenal insufficiency is often delayed due to the insidious onset and nonspecific symptoms (weakness, fatigue, musculoskeletal pain, weight loss, abdominal pain, depression, and anxiety). Some patients are misdiagnosed as having psychiatric illness (e.g., nervous anorexia) ( 28 ) or gastrointestinal disease ( 1 , 27 ).

### Incidence and mortality of AC

Although patient-initiated stress dosing seems to be effective to avert AC, the incidence of these events remains elevated. Two large retrospective postal survey studies comprising 1,287 adult patients with adrenal insufficiency from Germany, the United Kingdom, Canada, Australia, and New Zealand evidenced an AC incidence of around 6.3-8/100 patient years ( 25 , 32 ).

Discouraging results were seen even among patients instructed about prevention of AC. A 2-year German prospective study (n = 423 patients) analyzed the occurrence of AC in patients with adrenal insufficiency who had received detailed written instructions on glucocorticoid stress dose adjustments. The AC incidence was 8.3/100 patient years. Approximately one in 12 patients with adrenal insufficiency will experience a life-threatening crisis in the coming year, and patients with a previous AC are at 2.8-fold risk to develop later episodes. The main precipitating factors were gastrointestinal diseases, other infectious diseases, emotional stress, and stressful events (e.g., major pain, surgery, strenuous physical activity, heat, and pregnancy). In 7% of AC episodes, a precipitating factor was not identified. Four out of 10 deaths were associated with AC, 0.5 AC-related deaths per 100 patient years, and the AC mortality was approximately 6% ( 33 ).

Rushworth and cols. also cited older age, a prior AC, the presence of autoimmune polyglandular syndromes, type 1 diabetes mellitus, and nonendocrine coexisting conditions such as asthma and cardiac disease as AC risk factors among patients with hypoadrenalism ( 26 ). Inadequate discontinuance of glucocorticoid therapy by the patient (or by an attending physician) is also a triggering factor of AC ( 27 ).

Failure of adequate preventive measures and prompt diagnosis of AC by patients and physicians is common. A long-term Australian study analyzed all attendances between 2000 and 2017 of 56 PAI adult patients in a large regional referral center (252 attendances). Nearly half (45.2%, 114 out of 252) of the attendances were related to an infection. Only 2.8% (7 out of 252) used intramuscular (IM) hydrocortisone prior to presentation, and just 17.9% (45 out of 252) of the hospital presentations followed any form of stress dosing. The treating clinicians diagnosed 61 AC episodes (24.4%, 61out of 252). Among patients with a clinician-diagnosed AC, only 32.8% (20 out of 61) had used stress dosing before presentation ( 34 ).

Assuming an adrenal insufficiency prevalence of 82-144/million ( 1 ) and estimating the Brazilian population in 2020 at 211,755,692 people ( https://cidades.ibge.gov.br/brasil/panorama ), 17,363 to 30,492 Brazilians may be affected by adrenal insufficiency. As mortality from AC is estimated at 0.5/100 patient years ( 33 ), 868 to 1,524 deaths from AC are expected in Brazil in the coming decade. These data represent an alarming context regarding morbimortality and costs for public health systems, mainly because adrenal crisis is a preventable condition.

Among children, the incidence and mortality of AC is similar to that in adult patients: 5-10 episodes/100 patient years, with 1 death in every 200 episodes of AC ( 35 ).

Among CAH patients, AC is common even after the neonatal period. In a cross-sectional questionnaire-based study of 122 CAH patients, the AC frequency was 5.8/100 patient years in the whole group and 4.9/100 patient years after correction for neonatal SW crisis, with no difference in incidence between males and females. AC episodes mostly occurred during childhood, with one-third occurring in the first year and more than 70% within the first 10 years of life. Still, 20% of adrenal events were observed in adults. An age-related pattern was observed, with respiratory infections being the main trigger in early childhood, whereas gastrointestinal infections were the main cause at older ages. In this study, the median time for recognition of the first AC symptoms was one day, even in chronic, well-informed patients ( 36 ).

### Management and treatment of AC

Suspected AC requires immediate therapeutic action ( 27 ). In acute sick patients with clinical signs suspected of AC, treatment should not be delayed awaiting test results. Before hydrocortisone administration, a single basal adrenocorticotropic hormone (ACTH) and cortisol sample collection, at any time of the day, is essential for the diagnosis ( 2 ).

For adults, immediate parenteral injection of hydrocortisone (100 mg), as well as a fluid restoration with 1,000 mL of isotonic saline within the first hour, should be performed in cases of suspected AC. Subsequently, 200 mg of hydrocortisone should be administered during the first 24 hours (6 hourly injections or continuous iv administration). During the following day, the hydrocortisone dose should be reduced to 100 mg and, afterward, switched to a double oral glucocorticoid regimen depending on the clinical state. Prednisolone (25 mg as a bolus, followed by two 25 mg doses, for a total of 75 mg in the first 24 hours; thereafter, 50 mg every 24 hours) is an alternative if hydrocortisone is unavailable; dexamethasone should be avoided, only being given (4 mg every 24 hours) if no other glucocorticoid is available ( Table 1 ) ( 2 , 26 , 27 , 37 – 39 ).

**Table 1:** Management of adrenal crisis

Topic	Treatment
1	Volume expansion: 1,000 mL iv isotonic saline within the first hour. Children: 20 mL/kg isotonic saline. Can repeat up to a total of 60 mL/kg within 1 h for shock.
2	100 mg iv hydrocortisone followed by 200 mg/d hydrocortisone as a continuous infusion for 24 h or every 6 h, reduced to 100 mg/d hydrocortisone the following day. Children: 50-100 mg/m^2^ iv hydrocortisone bolus followed by 50-75-100 mg/m^2^/d hydrocortisone every 6 h or 2-3 mg/m^2^/h as a continuous infusion for 24 h. *Alternative simpler scheme of bolus stress doses of hydrocortisone: children < 15 kg (0-2 years), 25 mg; 15-25 kg (2-6 years), 50 mg; and > 25 kg (>6 years), 100 mg.
3	For hypoglycemia: 0.5-1 g/kg dextrose or 2-4 mL/kg of 25% dextrose solution (maximum single dose 25 g) infused slowly at a rate of 2 to 3 mL/min. Alternatively, 5-10 mL/kg of 10% dextrose solution for children < 12 years old
4	Cardiac and hemodynamic monitoring. Low heparin doses. Antibiotics if necessary.
5	Switch to oral regimen depending on clinical state.

Adapted from: Bornstein et al. ( 2 ), Allolio ( 27 ), Miller et al. ( 35 ), Nowotny et al. ( 39 ) and Shulman et al. ( 40 )*.

For children, 50-100 mg/m^2^ of hydrocortisone as an initial stress dose and fluid restoration with 20 mL/kg of isotonic saline within the first hour should administered in cases of suspected AC ( 35 ). The European Reference Network on Rare Endocrine Conditions (EndoERN) suggests an alternative scheme of hydrocortisone bolus stress doses based on the patient's age and weight: children < 15 kg (0-2 years), 25 mg; 15-25 kg (2-6 years), 50 mg; and > 25 kg (>6 years), 100 mg, as in adults ( 39 ). Subsequently, 50-75-100 mg/m^2^/day of iv hydrocortisone (6 hourly injections) or 2-3 mg/m^2^/hour (continuous iv) should be administered during the first 24 hours and reduced to 50 mg/m^2^ during the following day. Fludrocortisone is not required acutely due to the mineralocorticoid effect of high hydrocortisone dosage. Depending on the clinical state, the hydrocortisone should be switched to an oral regimen, starting with 30-50 mg/m^2^/day and reduced gradually to maintenance doses. In this moment, fludrocortisone 0.05-0.1 mg/day should be provided (2,35,39-41). As the stress doses are empirical and not based on controlled clinical trials, the recommended glucocorticoid stress doses vary in pediatric patients. Further studies are needed to define the ideal stress dose in pediatric patients, because both under- and overdoses are harmful to patients.

The factor must be investigated and treated in all patients with AC. Sodium and potassium levels should be monitored and iv dextrose should be administered in cases of hypoglycemia, more common in children. Antibiotic therapy, admission to an intensive care unit, and administration of a low dose of heparin should be considered ( 26 , 27 , 37 ).

The AC approach is often effective, with patient recovery within 24 hours. If recovery does not occur, two possibilities must be considered: Either there is another cause for the patient's serious impairment or the patient has reached “a point of no return” when even optimum care will no longer avert death from AC ( 27 ).

### Prevention of AC

Adrenal insufficiency patients, their families, and their caregivers should be constantly informed that adherence to continuous glucocorticoid therapy throughout life and adequate adjustment of glucocorticoid doses in stressful situations are essential to avert AC and death. Fever > 38 °C (100.4 °F), intercurrent illness with emesis, prolonged or voluminous diarrhea, infectious disease requiring antibiotics, acute trauma requiring medical intervention (e.g., fracture), and anesthesia-associated surgical procedures are considered stressful conditions. No randomized controlled studies have evaluated glucocorticoid dose requirements during stressful situations in either adults or children. Therefore, glucocorticoid doses are typically based on the general acceptance that cortisol levels rise 2 to 3 times during maximally stressful situations and on the severity and duration of the stressor or illness ( 2 , 27 , 35 ).

During fever, oral hydrocortisone replacement doses should be doubled (>38 °C) or tripled (>39 °C) until recovery (usually 2 to 3 days), and the consumption of electrolyte-containing fluids should be encouraged as tolerated. As soon as recovery occurs, the doses should be rapidly (within 1 to 2 days) reduced to the standard replacement doses. If oral medication is not tolerated (vomiting, diarrhea, or trauma), 100 mg of hydrocortisone for adults (50 mg/m^2^ for children), IV, IM, or subcutaneous (SC), should be either self-administered or administered by a physician. It may be necessary to repeat this dose. Health care professionals should be involved early for clinical assessment. Mineralocorticoid replacement is not required if the hydrocortisone dose exceeds 50 mg/24 h due to the mineralocorticoid effect of high hydrocortisone dosage ( Table 2 ) ( 2 , 27 , 33 , 42 ).

**Table 2:** Stress doses of hydrocortisone to prevent and/or treat adrenal crisis

Clinical Condition	Treatment
Fever and illness - home management	Hydrocortisone replacement doses doubled (>38 °C) or tripled (>39 °C) until recovery and increased consumption of electrolyte-containing fluids as tolerated
Oral intake prevented by vomiting or trauma	Adults: 100 mg IM or SC hydrocortisone (children: 50 mg/m^2^)
Minor to moderate surgical stress	Doubled or tripled hydrocortisone oral doses or 25-75 mg/24 h iv hydrocortisone (usually 1 to 2 d) in adults (50 mg/m^2^ in children)
Major surgery with general anesthesia, trauma, delivery, or disease that requires intensive care	100 mg IV hydrocortisone followed by 200 mg/d hydrocortisone as a continuous infusion for 24 h or every 6 h, reduced to 100 mg/d hydrocortisone the following day. Children: 50-100 mg/m^2^ hydrocortisone bolus at the induction of anesthesia, followed by 50-100 mg/m^2^/d hydrocortisone divided q 6 h. Decrease to 50 mg/m^2^/d hydrocortisone on the second day and 25 mg/m^2^/d on the third day.

Adapted from: Allolio ( 27 ), Miller et al. ( 35 ) and Shulman et al. ( 40 ).

Concerning surgical stress, it is estimated that adults secrete 75-100 mg of cortisol/day in response to major surgery and 50 mg/d in response to minor surgery ( 42 ). Prior to minor or moderate surgical stress, doubled or tripled hydrocortisone replacement oral doses or 25-75 mg/24 h IV hydrocortisone is recommended for adults (usually 1 to 2 days) and 50 mg/m^2^/24 h for children during the procedure. Prior to major surgery with general anesthesia or during delivery (4 cm cervix dilation and/or contractions every 5 min for the last hour), trauma, or disease that requires intensive care, the parenteral hydrocortisone management should be performed as in AC, for both adults and children ( Table 2 ) ( 2 , 40 ). In SW CAH children, we suggest the oral administration of the usual mineralocorticoid doses around 4 hours before the start of surgery (unpublished data).

### Patients’ education about AC prevention

AC in patients with known PAI and CAH is best prevented by educating patients and caregivers on the precocious identification of AC and the glucocorticoid dose adjustment during stressful situations ( 27 ).

However, it has been shown that a high percentage of patients (46%) were not sufficiently skilled in steroid management with physical stress ( 43 ). The most effective strategy to check if the patients are regularly on such therapy is by asking them if they are aware of how important the medication is, how it must be taken daily, and how it should be taken in different stressful situations ( Table 2 ). If the patient fails, information should be provided again, and all doubts should be cleared.

**Figure 1:**
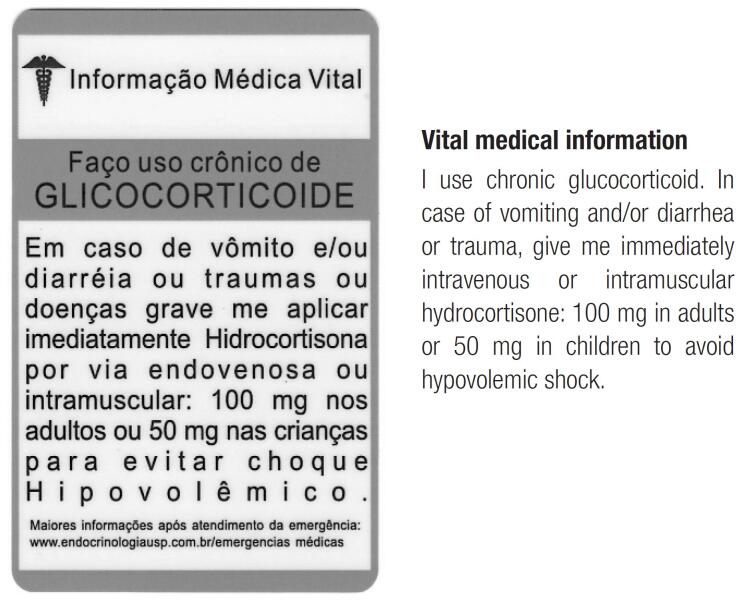
Adrenal crisis treatment card.

Besides that, an AC prevention card, with a medical alert to inform patients and health professionals about the acute hydrocortisone injection (IM or IV) in stressful situations, should be offered to the patients and caregivers ( Figure 1 ) ( 37 , 38 , 44 ). The patients should carry the card with them constantly, which should be checked by the physician at every medical appointment. This card should be available for all patients with adrenal insufficiency to avoid hypovolemic shock and death. The cost is reasonable (around US$ 40.00 for 1,000 cards in Brazil) and should ideally be supported by the hospital.

In addition, every patient should carry an emergency glucocorticoid injection kit consisting of 100 mg of hydrocortisone for parenteral or SC administration. This kit urges the first parenteral administration of glucocorticoids even before the patient can get in touch with a doctor or reach an emergency service ( 2 ). In Brazil, this kit is not commercially available yet, and efforts are being made to enable pharmaceutical companies to provide it here, because injectable hydrocortisone is only available in hospitals.

In conclusion, thus, there is an increased mortality among PAI and CAH patients linked to episodes of AC, which occur frequently during infections and stress conditions. If the AC is promptly identified and managed, morbidity and mortality is reduced. Unfortunately, as the clinical features of imminent AC are often nonspecific, the glucocorticoid stress dose is frequently delayed. Effective prevention of AC remains a major challenge in the care of these patients. Patients’ and caregivers’ education, the emergency card, and the glucocorticoid kit are essential to preventing AC and reducing the morbimortality.
